# Irradiation-injured brain tissues can self-renew in the absence of the pivotal tumor suppressor p53 in the medaka ( *Oryzias latipes* ) embryo

**DOI:** 10.1093/jrr/rrv054

**Published:** 2015-09-25

**Authors:** Takako Yasuda, Yoshitaka Kimori, Kento Nagata, Kento Igarashi, Tomomi Watanabe-Asaka, Shoji Oda, Hiroshi Mitani

**Affiliations:** 1 Department of Integrated Biosciences, Graduate School of Frontier Sciences, Tokyo University, Bioscience Building 102, Kashiwa, Chiba 277–8562, Japan; 2 Department of Imaging Science, Center for Novel Science Initiatives, National Institutes of Natural Sciences, Okazaki, 444-8787, Japan

**Keywords:** p53, medaka, self-renewal, developing brain, radiation, histology

## Abstract

The tumor suppressor protein, p53, plays pivotal roles in regulating apoptosis and proliferation in the embryonic and adult central nervous system (CNS) following neuronal injuries such as those induced by ionizing radiation. There is increasing evidence that p53 negatively regulates the self-renewal of neural stem cells in the adult murine brain; however, it is still unknown whether p53 is essential for self-renewal in the injured developing CNS. Previously, we demonstrated that the numbers of apoptotic cells in medaka ( *Oryzias latipes* ) embryos decreased in the absence of p53 at 12–24 h after irradiation with 10-Gy gamma rays. Here, we used histology to examine the later morphological development of the irradiated medaka brain. In p53-deficient larvae, the embryonic brain possessed similar vacuoles in the brain and retina, although the vacuoles were much smaller and fewer than those found in wild-type embryos. At the time of hatching (6 days after irradiation), no brain abnormality was observed. In contrast, severe disorganized neuronal arrangements were still present in the brain of irradiated wild-type embryos. Our present results demonstrated that self-renewal of the brain tissue completed faster in the absence of p53 than wild type at the time of hatching because p53 reduces the acute severe neural apoptosis induced by irradiation, suggesting that p53 is not essential for tissue self-renewal in developing brain.

## INTRODUCTION

Adult neu`ral stem cells (aNSCs) have the ability to generate new neural cells continuously and contribute to brain homeostasis, which is essential for plasticity and neural regeneration following brain damage. There have been a number of studies addressing the therapeutic potential of aNSCs after brain injury [ 1 , 2 , 3 ].

The tumor suppressor protein, p53, is a DNA-binding transcription factor encoded by *Trp53* . It induces apoptosis and cell cycle arrest in response to various genotoxic stresses to block the transmission of induced DNA mutations to progeny cells [ 4 ]. Several studies indicate that the loss of p53 function enhances cell proliferation in the self-renewal of aNSCs in the adult murine brain, suggesting that p53 acts to suppress neurogenesis among these stem cells [ 5 , 6 , 7 ]. However, it remains unclear whether p53 is involved in tissue self-renewal when the developing embryonic brain is damaged.

The medaka ( *Oryzias latipes* ) is an excellent model animal for studies on the developing central nervous system (CNS) of vertebrates, because the transparency and small size of embryos permits developing CNS morphology to be examined with considerable ease compared with mammalian embryos [ 8 ]. In addition, morphogenesis in the medaka embryonic brain is slower than in zebrafish ( *Danio rerio* )—another popular laboratory model fish—and can provide more detailed information about the effects of irradiation on the developing brain [ 9 ]. In our previous study, medaka embryos were irradiated at Developmental Stages 28–30 [ 10 ], when neural cells proliferate rapidly, especially in the optic tectum (OT), and radiation-induced apoptotic cells were localized mainly in the margin of the OT up to 30 h after irradiation. After this, the numbers of acridine orange (AO)–positive apoptotic cells decreased gradually and disappeared by 48 h after irradiation [ 11 ]. When embryos of the p53-deficient medaka [ 12 ] were irradiated, AO-positive apoptotic neurons also appeared in the retina and the margin of the OT; however, there were many fewer apoptotic neurons than in wild-type (wt) embryos. As shown in our previous study, the irradiated wt and p53-deficient embryos hatch normally and develop with no apparent abnormalities in their behaviors when they mature [ 13 , 14 ].

Here, we used histology to examine tissue self-renewal in irradiated neural tissue up to the hatching period and found that the p53 protein was not essential for this. We also employed a new evaluation method, Voronoi tessellation [ 15 ], for quantitative analysis of randomness of the spatial distribution of neurons in the irradiated OT cells on histological sections to evaluate the effects of ionizing radiation on neurogenesis.

## MATERIALS AND METHODS

### Fish and embryos

This study was conducted according to The University of Tokyo Animal Experiment Enforcement Rule.

Medaka stocks of the Hd-rR inbred strain, established from the southern population [ 16 ], were kept in the laboratory. A strain of p53-deficient fish originally generated by the Targeting Induced Local Lesions in Genomes method, with a genetic background of the Cab strain [ 12 ] were backcrossed four times with Hd-rR strain fish to establish a p53-deficient strain with an Hd-rR genomic background. The fish were kept at 26–28°C under a 14 h light and 10 h dark cycle, and fed on a powdered diet (TetraFin; Tetra Werke Co., Melle, Germany) and live brine shrimp ( *Artemia franciscana* ) three times daily.

Egg clusters were collected every morning from female fish, and the collected eggs were isolated by rubbing them between two small pieces of paper towel to remove filaments on the chorion. Then, the isolated eggs were incubated in a petri dish filled with 7 ml of tap water containing 10 ^–5^ % (w/v) methylene blue at 26–28°C. The developmental stages of the embryos are described according to Iwamatsu (2004) [ 10 ]. The schematic image of medaka embryonic development from fertilization to hatching was summarized as shown in supplementary Fig. S1 .

### Irradiation

We examined the effects of irradiation on developing brain in the somitogenesis period at Stages 29–30, (34–35-somite stages, 74–82 h after fertilization), when neuronal cell production and migration are in full swing, which correspond approximately to the early fetal stage of humans at ∼8–15 weeks post-ovulation [ 17 ]. Embryos at Stages 29–30, (34–35-somite stages, 74–82 h after fertilization) were irradiated by ^137^ Cs gamma-rays (10 Gy, Gammacell 3000 Elan; MDS Nordion, Ottawa, Canada) at a dose rate of 7.8 Gy/min at room temperature in a water-filled plastic tube.

### Quantitative real-time qPCR and whole-mount *in situ* hybridization

Cyclin-dependent kinase inhibitor 1A, p21, ap53-downstream gene, has been suggested to mediate p53-induced growth arrest triggered by DNA damage [ 18 ]. The sequence of the medaka gene encoding p21 was obtained from the Ensembl Genome Browser ( http://asia.ensembl.org/index.html ) database. Total RNA isolation from non-irradiated and irradiated embryos of wt and p53-deficient embryos, cDNA synthesis, and quantitative real-time qPCR were performed as described [ 19 ]. The primer pair p21 forward, 5′–CAACGTGGAGAAAACACCAG–3′, and reverse, 5′–CCATTCGTCGTTTAGCTTGG–3′, were used for quantitative real-time qPCR; and the primer pair p21 forward, 5′–ATGGCTGCTCCAAAGCGG–3′, and reverse, 5′–TCACTCGCCCGATTTCCG–3′, were used as probes for whole-mount *in situ* (WISH). The amplified p21 cDNA was cloned into pCR4-TOPO (Invitrogen, CA, USA), digested with Not I, and transcribed *in vitro* with T3 polymerase to prepare digoxigenin-labeled RNA probes for WISH. Medaka embryos at Developmental Stage 28 were anesthetized on ice, fixed in 4% (w/v) paraformaldehyde in 0.1 M phosphate buffer overnight at 0–4°C, dechorionated manually with fine tweezers and washed in PBS with 0.1% Tween20. The embryos were dehydrated in methanol and WISH was performed in accordance with the method as described [ 20 ].

### Observation of apoptosis by histology

Medaka embryos and hatched larvae were anesthetized on ice and fixed in 4% (w/v) paraformaldehyde in 0.1 M phosphate buffer overnight at 0–4°C, then dehydrated in an ethanol series, embedded in resin (Technovit 8100; Heraeus Kulzer, Wehrheim, Germany), and sectioned frontally into a complete series of serial sections (8-μm thick), as described [ 11 ]. The sections were Nissl stained with cresyl violet for light microscopy (BX50; Olympus, Tokyo, Japan).

### Quantitative histology of the spatial distribution of cells in the OT

The spatial distribution of OT cells in irradiated and non-irradiated embryonic brains was analyzed using Voronoi tessellation [ 15 ], which is a decomposition of 2D planes into independent polygons associated with each cell. The area of each Voronoi polygon corresponds to the area occupied by a single cell, and the size of the area depends on the relationships between neighboring cells. Therefore, a statistical analysis of the polygonal areas provides morphometric information about the spatial distribution pattern of the cells.

The basic image-processing procedure in this study is illustrated in Fig. 3 . The original image (Fig. 3 A) is a portion of a micrograph of a histological section of the OT region in the irradiated p53-deficient embryos. First, the regions of cell nuclei were segmented using a Top-hat filter based on rotational morphological processing [ 21 , 22 ] followed by automatic thresholding (Fig. 3 A–D). Next, the X–Y coordinates of the centroids of each nucleus were calculated (Fig. 3 E). Finally, the Voronoi tessellation of each microscope field was calculated from the coordinates (Fig. 3 F), and the areas of Voronoi polygons were computed. The variability of these areas characterizes the randomness of the spatial distribution pattern of the cells. We adopted the coefficient of variation (CV) for indexing the degree of variation of the polygon areas. The CV is defined as the ratio between the standard deviation of the polygon area and its mean. Generally, the CV value increases with increasing randomness of the spatial distribution pattern.

## RESULTS

### Tissue self-renewal in injured embryonic brain of wt and p53-deficient embryos after irradiation

**Fig. 1.**
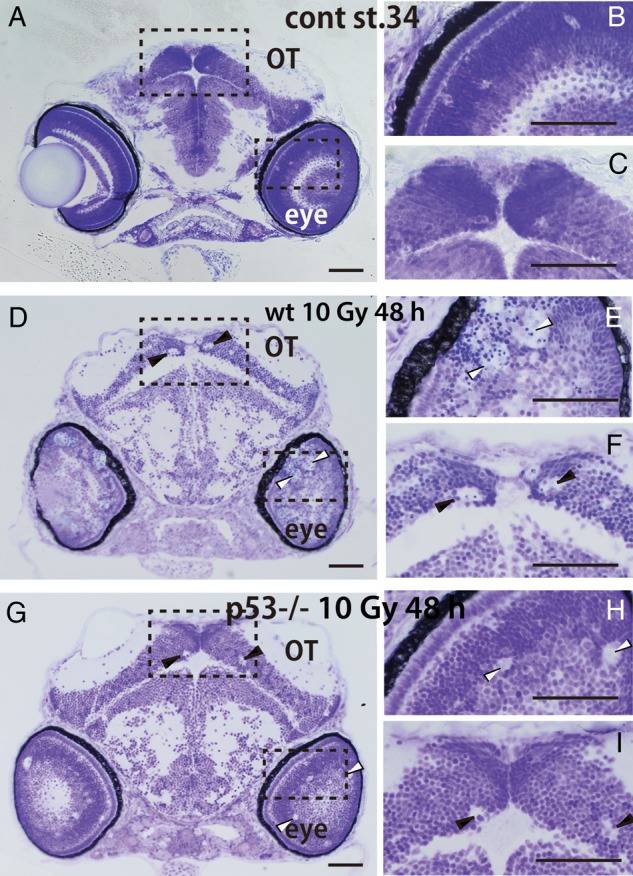
Frontal sections of irradiated embryonic brains at 48 h after irradiation (Stage 34). ( **A** ) Nissl-stained plastic section of a non-irradiated embryonic brain at the Developmental Stage 34 showed no round vacuoles in the optic tectum (OT) or developing retina. Higher magnifications of the eye and OT in the boxed areas in Fig. A are shown in **B** and **C** . ( **D** ) In the irradiated wild-type (wt) embryo at 48 h after irradiation showed many round vacuoles in the OT and developing retina. Higher magnification of the eye and OT in the boxed areas in D shows obvious round vacuoles including condensed nuclei in the retina (open arrowheads in **E** ) and in the margin of the OT (arrowheads in **F** ) of an irradiated wt embryo.( **G** ) In the irradiated p53-deficient embryonic brain, similar but much smaller and fewer vacuoles were present in the retina (open arrowheads in **H** ) and in the margin of the OT (arrowheads in **I** ) at 48 h after irradiation. Scale bars = 50 μm.

**Fig. 2.**
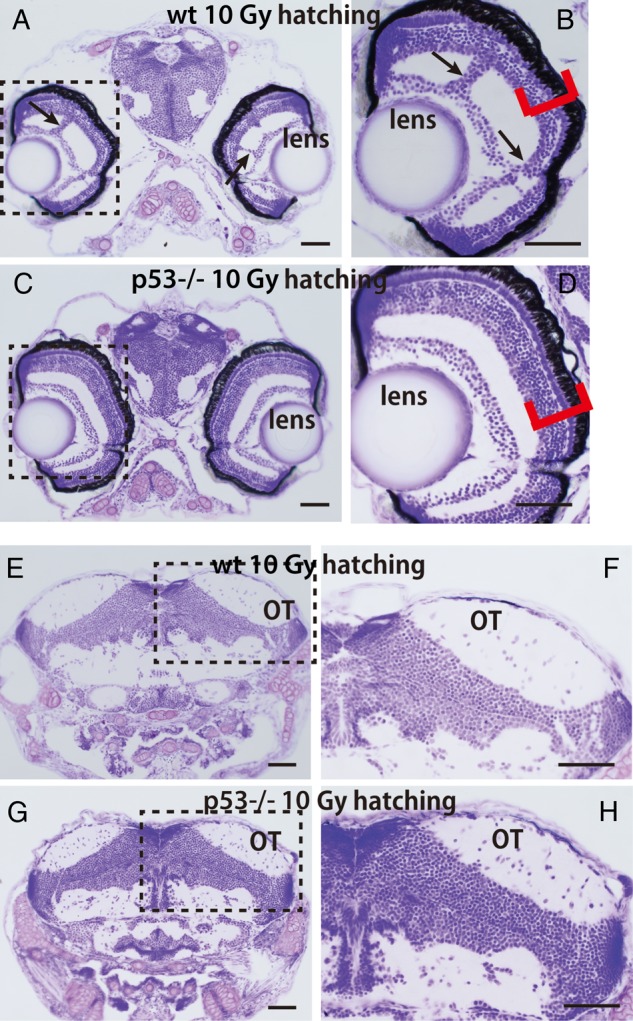
Histology of the eyes and OT of irradiated embryos at the hatching stage. Shown are Nissl-stained frontal plastic sections of the head region of an irradiated wt ( **A** ) and a p53-deficient hatching larva ( **C** ). ( **B** ) A higher magnification of the boxed area in A shows abnormal laminar arrangements in the retina (red brackets) and abnormal structures bridging layers of retinal neurons (arrows) in the irradiated hatching larvae. ( **D** ) A higher magnification of the boxed area in C shows well-ordered laminar arrangements in the retina. Frontal plastic sections are shown including the OT in irradiated wt embryos at hatching ( **E** ) and p53-deficient embryos at hatching ( **G** ). Higher magnifications of the boxed area in E ( **F** ) and of the boxed area in G ( **H** ) showed no abnormal cell arrangements in either of the irradiated OT. Scale bars = 50 μm.

**Fig. 3.**
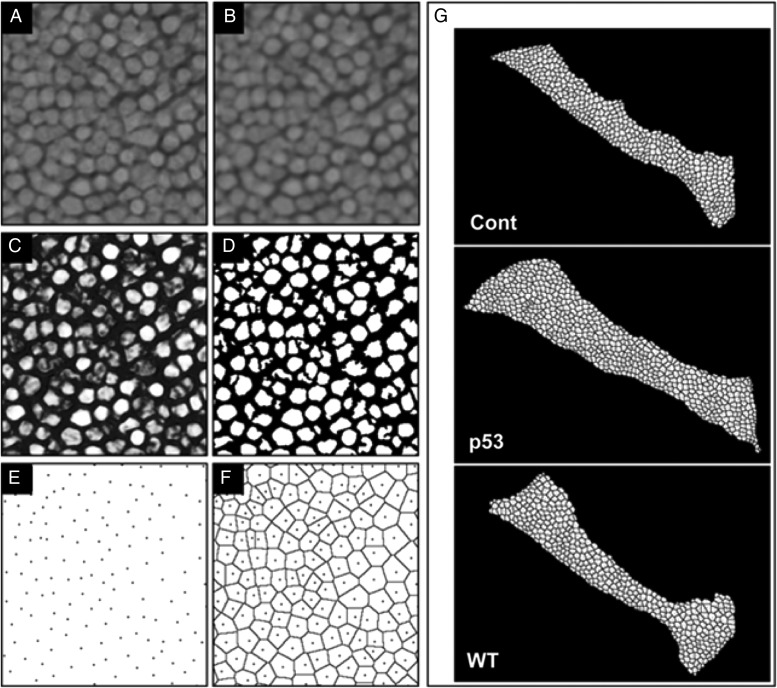
Voronoi tessellation of OT regions for quantitative analysis of the spatial distribution of cells. The original image of the nuclei of neurons in the OT ( **A** ) was smoothed by Gaussian blurring to reduce the effect of noise ( **B** ). Then, a morphological Top-hat filter was applied to the smoothed image ( **C** ), and each nuclear region was segmented after automatic thresholding ( **D** ). Centroids of each nuclear region ( **E** ) were extracted and are shown as dots. The polygonal areas are drawn in ( **F** ) and Voronoi tessellation computed from the centroid positions, and the results of Voronoi tessellation of OT regions are presented for control, irradiated p53-deficient and irradiated wt embryos (cont., p53 and WT in G, respectively).

As reported previously [ 13 ], gamma-ray irradiation (10 Gy) induced neural apoptosis in the marginal area of OT within 12 h in both wt and p53-deficient embryos ( Supplementary Fig. S2 ). The clusters of AO-positive apoptotic cells disappeared at 18 h after irradiation in the OT of the p53-deficient embryos. In contrast, they remained up to 36 h after irradiation in the irradiated wt embryos and then disappeared completely at 48 h after irradiation—at approximately Stage 34, 5 days after fertilization—while degenerating nuclei, in which were observed condensed nuclei inside the round vacuoles, remained in the retina (open arrowheads in Fig. 1 D, E) and in the margin of the OT (arrowheads in Fig. 1 D, F). Time course appearance of degenerating apoptotic neurons in vacuoles has been demonstrated by electron microscopic observations in our previous report [ 13 ]. These observations confirmed that vacuoles were a part of the phagosome of microglia for clearing apoptotic debris. At this time-point, few condensed nuclei were present in the vacuoles of irradiated p53-deficient embryos in the retina (open arrowheads in Fig. 1 G, H) and in the OT (arrowheads in Fig. 1 G, I) because their considerably reduced induction of apoptotic neurons would have been digested already. The appearance of vacuoles in p53-deficient embryos was similar with wt embryos; however, they were much smaller and fewer than those of wt embryos. These round vacuoles were never observed in non-irradiated wt embryos at Stage 34 (2 days after irradiation; 5 days after fertilization) (Fig. 1 A–C). At the time of hatching (6 days after irradiation; 9 days after fertilization), the vacuoles with degenerating nuclei disappeared completely in the retina and the marginal area of the OT in the wt and p53-deficient larvae (Fig. 2 ). However, in the irradiated wt larvae, the laminar arrangement of the retinal neurons was obviously disorganized (red bracket in Fig. 2 B), and abnormal bridging structures among the layers of retinal neurons were present ( *n* = 3; arrows in Fig. 2 A, B). These were never observed in the irradiated p53-deficient larvae (Fig. 2 C, D). Furthermore, there were no apparent histological abnormalities in the OT of either wt or p53-deficient larvae (Fig. 2 E–H); however, analysis of the randomness in spatial distribution of the neural cells in the OT using Voronoi tessellation gave a high CV value of 0.351 for the irradiated wt hatching larvae, indicating that the neurons of the OT in the irradiated wt embryonic brain were arranged in a more random manner than in the non-irradiated wt embryos, and that self-renewal of neural tissues had not been completed at the time of hatching in these larvae. In contrast, the CV values were equal in the irradiated p53-deficient larvae and to those in the non-irradiated control larvae (0.317 for both, Fig. 3 G and Table 1 ), strongly suggesting that self-renewal of the irradiated brain tissue was complete at the time of hatching in the absence of p53.

**Table 1.** Statistics of Voronoi polygonal areas

	control	p53 ^−/−^	wt
Number	889	1704	1398
Mean (μm ^2^ )	184.864	197.706	206.672
S.D.	58.632	62.644	72.492
CV	0.317	0.317	0.351

Numbers represent the means of counted cells and mean (μm ^2^ ) represents the mean areas examined in the OT (three images were examined for each titles). The coefficient of variation (CV) was defined as the ratio between the standard deviation (SD) of the polygonal areas and their mean values.

### Absence of prominent induction of p21 expression by irradiation in p53-deficient embryos

**Fig. 4.**
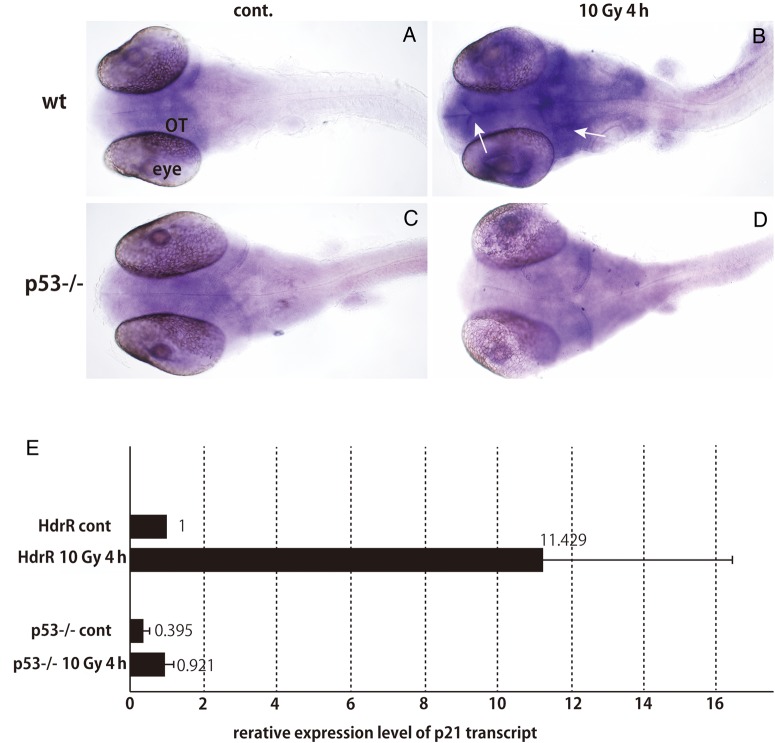
Effects of p21 upregulation in irradiated wt and p53-deficient medaka embryos 4 h after irradiation demonstrated by whole-mount *in situ* hybridization (WISH) and quantitative real-time qPCR. Gamma-ray irradiation increased p21 expression in the margin of the OT by 4 h after irradiation (white arrows in **B** ), but only in the wt embryos. In contrast, the p21 expression level in the irradiated p53-deficient embryo ( **D** ) was the same as in the non-irradiated embryos ( **A** , **C** ). This upregulation (11-fold) was confirmed by quantitative real-time qPCR in the wt embryos after irradiation. The p21 expression level was lower in the p53-deficient embryos than in the non-irradiated wt embryos, even after being irradiated ( **E** ). Error bars represent standard deviation of the mean ( *n* = 3).

To confirm the p53 protein is not activated in p53-deficient embryos, the expression of the cyclin-dependent kinase inhibitor 1A, p21, was examined by WISH and quantitative real-time qPCR. The expression of p21 was induced especially in the margin of the OT in wt embryos at 4 h after irradiation (arrows in Fig. 4 B), implicating that irradiation upregulated the expression of p21, especially in neuronal cells in wt embryos. In contrast, p21 upregulation was completely absent in the irradiated p53-deficient embryos (Fig. 4 D, E). Quantitative real-time qPCR demonstrated the prominent increase (11-fold) in p21 transcripts after irradiation in the wt embryos, whereas the p21 expression level was lower than in the non-irradiated wt embryos, even after irradiation in the p53-deficient embryo (Fig. 4 E). These results strongly suggest that the function of p53 was completely lacking in the p53-deficient embryos.

## DISCUSSION

In our previous studies, medaka embryos irradiated at Developmental Stages 28–30 [10], 3 days after fertilization, showed a remarkable increase in apoptotic cells in the margin of the OT; then apoptotic cells decreased gradually and disappeared completely by 48 h after irradiation [ 13 ]. However, the later process of tissue repair in the irradiated embryonic brain remained to be investigated. Here, we examined tissue self-renewal in the neural system using irradiated embryos of the wt and the p53-deficient medaka.

At 48 h after irradiation, there were tissue abnormalities such as large vacuoles, believed to be the phagosomes of microglia clustering the apoptotic neuronal cells—as reported previously in zebrafish [ 23 ]—in the irradiated wt and p53-deficient brains. The phagosomes of microglia could not be visualized by AO staining at 48–49 h after irradiation [ 11 ]. While it is not clear how AO accumulates in apoptotic cells but not in dead cells [ 24 ], the present results confirmed that AO-positive cells changed to form apoptotic cell debris and lost their AO staining in the phagosomes of microglia at 48–49 h after irradiation. However, histological observations showed that they remained as masses of degenerating nuclei and dead cells, which has been also confirmed by time-course electron microscopic observations, as shown in our previous paper [ 13 , 25 ].

At the hatching stage (6 days after irradiation; 9 days after fertilization), vacuoles had disappeared in the retina and OT of irradiated wt and p53-deficient embryos; however, abnormal neuronal arrangements and abnormal bridging structures in the retina remained only in the wt hatching larvae (Fig. 2 A, B). Furthermore, Voronoi tessellation analysis demonstrated that neural cells in the OT of irradiated wt embryos were arranged in a more random manner than in non-irradiated embryos. Voronoi tessellation analysis was used to evaluate randomness in the spatial distribution of OT neural cells, and was applied here for histology of the CNS for the first time as far as we know. The more random arrangement of OT neural cells in the wt larvae would never be elucidated using conventional histology. These results suggest that the irradiated wt embryos might have some defective visual capacity, although they hatch normally and develop with no apparent abnormalities in their behaviors when they mature as shown in our previous report [ 13 , 14 ]. So, it would be necessary to examine their behavior carefully to clarify whether the tissues had self-renewed normally after hatching.

The irradiation-damaged neural tissues underwent self-renewal, and no structural abnormalities were found at the time of hatching (6 days after irradiation; 9 days after fertilization) in the absence of p53, suggesting a possibility that p53 works as not only useful but rather detrimental when immature neuronal cells were severely damaged. In the p53-deficient embryos, expression of p21 was suppressed, even after irradiation, confirming that the function of p53 as a transcriptional factor was almost completely blocked. In contrast, the damaged tissues also self-renewed in the presence of p53; however, tissue self-renewal was not complete by the time of hatching. There is accumulating evidence that p53 might suppress the self-renewal of aNSCs in the adult murine brain [ 5 , 6 , 7 ], suggesting that p53 regulates tissue self-renewal negatively in adult tissues. The present results seem to be consistent with these reports, although p53 might play a role in promoting tissue self-renewal through its complex functions in cell cycle regulation, as reported by Isoe *et al.* [ 26 ], who demonstrated that p53 positively regulates neurogenesis in the adult medaka brain. As demonstrated by our previous report [ 13 ] and as shown in Fig. 1 (see also Supplementary Fig. S2 ), gamma-ray irradiation induced fewer apoptotic cells in the p53-deficient embryonic brain. Tissue self-renewal was not complete at the time of hatching in the presence of p53, because the tissues were more severely injured by p53-dependent apoptosis in the wt embryo. The findings reported here suggest that the loss of p53 functions could help minimize tissue damage after irradiation, although cells with severely damaged DNA would survive because of cessation of apoptotic cell death in the absence of p53, and this could increase the risk of carcinogenesis later in the adult [ 27 ]. In future, we need careful observations if p53 works as detrimental or useful effects in later life of irradiated wt and p53-deficient medaka.

Finally, we emphasize the usefulness of p53 studies using medaka embryos *in vivo* for clarifying the process of self-renewal for damaged tissue repair during development, compared with studies using mouse models. One of the advantages of the medaka model is that apoptotic cells can be observed *in vivo* throughout the whole brain because of its small size and transparency. Histological abnormalities in the whole brain of medaka can be identified easily because fewer than 50 serial sections can cover the whole embryonic brain, and 3D image construction is possible. Such studies would be much more laborious in mouse embryos.

## FUNDING

This research was partly supported by a Grant-in-Aid for Scientific Research (C) Numbers 22510056 and 25514002 to T.Y., a Grant-in-Aid for Scientific Research (B) Number 24310039 to S.O. and a Grant-in-Aid for Scientific Research (S) Number 25220102 to H.M. from the Ministry of Education, Culture, Sports, Science, and Technology of Japan. Funding to pay the Open Access publication charges for this article was provided by a Grant-in-Aid for Scientific Research (C) Numbers 25514002 to T.Y.

## Supplementary Material

Supplementary Data
