# Supplementary material for: Irradiation-injured brain tissues can self-renew in the absence of the pivotal tumor suppressor p53 in the medaka ( Oryzias latipes ) embryo
Source: J Radiat Res. 2015 Sep 25;57(1):9–15. doi: 10.1093/jrr/rrv054 (PMC4708913; doi:10.1093/jrr/rrv054)

Supplemental Fig. S1 The schematic image of medaka embryonic development from fertilization to hatching Each images showed the embryonic development at fertilization, blastula, gastrula, embryogenesis, late embryo and hatching.


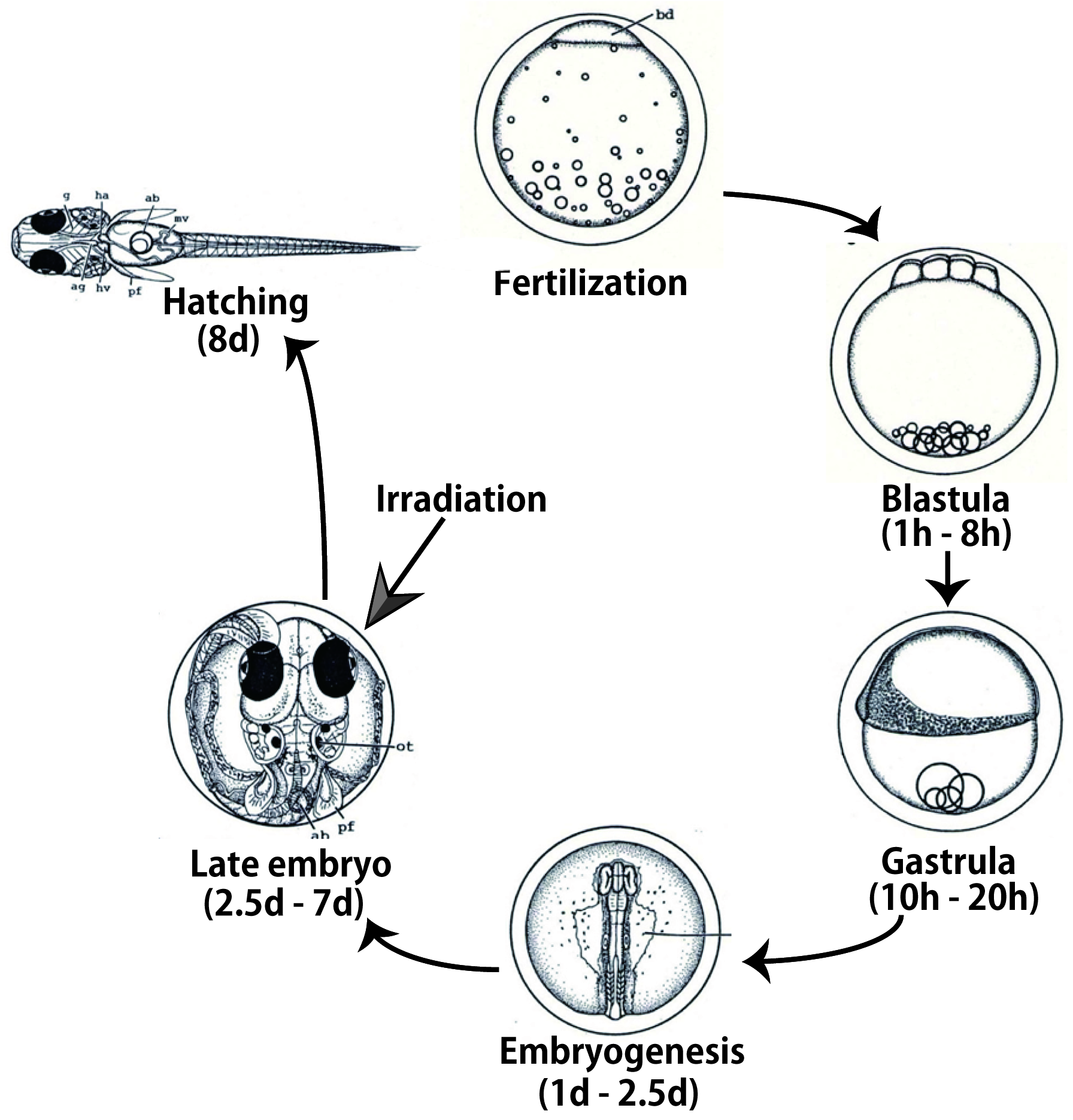


Supplemental Fig. S2. Radiation induced apoptotic cells in wild type (wt) and p53-deficient embryos were stained with acridine orange (AO) and examined by light microscopy at 12 hours after irradiation. (A) Schematic diagram illustrating the structure of the medaka embryonic brain (stage 30). (B) AO-stained apoptotic cells in wt embryos(arrows in B) at 12 h after irradiation. (C) AO-stained apoptotic cells in the p53-deficient embryos (arrows in C) at 12 h after irradiation. Frontal plastic sections including Nissl-stained eyes showed radiation-induced apoptotic cells in the irradiated wt embryos at 12 h after irradiation (arrows in E) and in the irradiated p53-deficient embryos (arrows in F), in contrast to no apoptotic cells in control embryonic brains (D). Highly magnified views of the boxed areas in D, E and F correspond to, H and I. Scale bars = 50 μm.


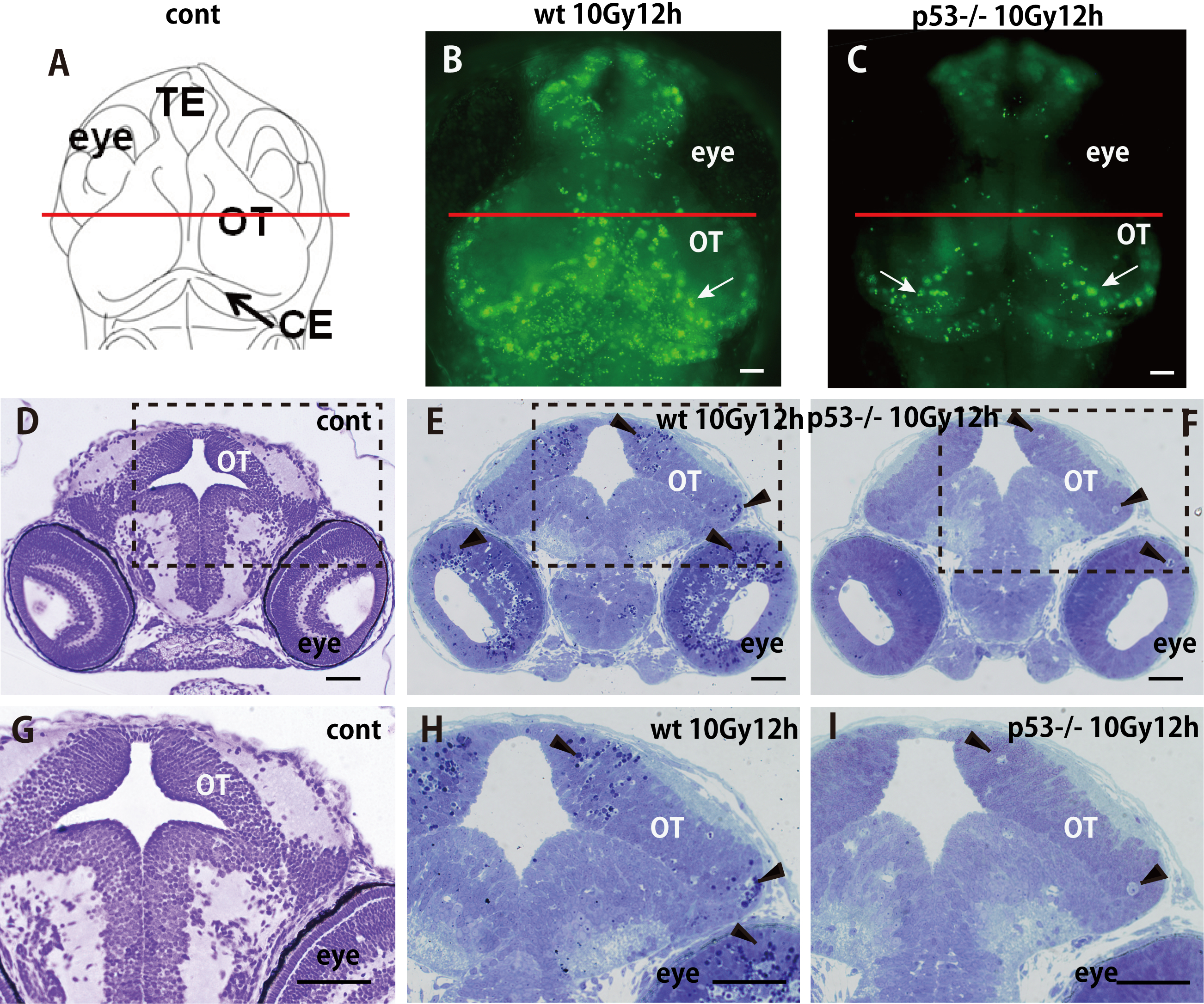

Supplement: Supplementary Data [file rrv054_Supplementary_Data.zip › rrv054supp.docx]
